# Supplementary material for: Genetic characterization of primary and metastatic high-grade serous ovarian cancer tumors reveals distinct features associated with survival
Source: Commun Biol. 2023 Jul 3;6:688. doi: 10.1038/s42003-023-05026-3 (PMC10318066; doi:10.1038/s42003-023-05026-3)
Supplement: Supplementary file 2 — Supplementary Information-New [file 42003_2023_5026_MOESM2_ESM.pdf]

SUPPLEMENTARY TABLE TITLES AND LEGENDS

Supplementary Table 1: Tumor Sample WES coverage

Exome Sequencing Coverage			
**Patient ID**	**Normal**	**Primary**	**Metastatic**
ST_001	148	244	251
ST_002	136	258	309
ST_004	79	388	225
ST_007	139	231	274
ST_009	97	325	267
ST_011	113	179	180
ST_013	131	365	234
ST_014	101	248	246
ST_015	259	309	559
ST_016	114	266	271
ST_017	107	309	272
ST_018	146	235	316
ST_019	157	279	295
ST_020	131	279	390
ST_021	145	231	306
ST_022	127	264	350
ST_023	142	384	309
ST_025	150	272	356
ST_026	112	250	266
ST_027	237	258	266
ST_028	117	279	339
ST_029	131	230	279
ST_030	65	331	139
LT_031	133	244	442
LT_033	226	300	316
LT_034	169	428	248
LT_035	142	278	355
LT_036	210	299	332
LT_037	213	263	336
LT_038	120	344	288
LT_039	175	389	218
LT_041	167	275	319
LT_042	186	338	181
LT_043	90	439	364
LT_044	241	331	278
LT_045	138	396	344
LT_046	187	283	331

Exome sequencing coverage for each patient’s normal, primary, and metastatic tumor. Coverage is expressed as the number of times a base would be covered by sequencing reads.

Supplementary Table 2 BRCA1 Somatic Mutations

Patient ID	Clonal Variant	Variant Type	Nucleotide Position	Amino Acid Change	Primary VAF	Metastatic VAF	ClinVar Clinical Significance
ST_009	yes	missense	c.5344A>C	p.F1782V	72.48	46.46	not provided
ST_015	yes	frame-shift deletion	c.2419	p.A807fs	36.96	42.76	pathogenic
LT_039	no	missense	c.3419C>G	p.S1140T	56.67	NA	uncertain significance
LT_042	yes	frame-shift deletion	c.2284	p.R762fs	92.04	75.77	pathogenic

All BRCA1 somatic mutations identified in tumor cohort for WES with a VAF>30. Nucleotide position and amino acid changes are based on GRCh37.

Supplementary Table 3 BRCA1 Germline Mutations

Patient ID	Variant Type	Nucleotide Position	Amino Acid Change	VAF	ClinVar Clinical Significance
ST_021	missense	c.4798G>C	p.P1600A	53.77	conflicting interpretations of pathogenicity
ST_027	missense	c.1487C>T	p.R496H	43.96	benign
ST_027	missense	c.455A>G	p.L152P	59.51	conflicting interpretations of pathogenicity
ST_029	missense	c.4931G>C	p.A1644G	51.67	pathogenic/likely pathogenic
LT_031	nonsense	c.2035T>A	p.K679*	57.81	pathogenic
LT_031	missense	c.736A>C	p.L246V	55.29	benign
LT_037	missense	c.5396T>C	p.D1799G	42.86	conflicting interpretations of pathogenicity
LT_044	nonsense	c.2138G>C	p.S713*	37.01	pathogenic

All BRCA1 germline mutation identified in patient cohort from WES with a VAF>30. Nucleotide position and amino acid changes are based on GRCh37.

Supplementary Table 4 BRCA2 Germline Mutations

Patient ID	Variant Type	Nucleotide Position	Amino Acid Change	VAF	ClinVar Clinical Significance
LT_039	missense	c.3568C>T	p.R1190W	47.89	benign

All BRCA2 germline mutations identified in patient cohort from WES with a VAF>30. Nucleotide position and amino acid changes are based on GRCh37.

Supplementary Table 5 Somatic TP53 mutations

Patient ID	Clonal Variant	Variant Type	Nucleotide Position	Amino Acid Change	p53 Protein Domain	Primary VAF	Metastatic VAF
ST_001	yes	missense	c.536T>C	p.H179R	DNA-binding domain	55.70	23.85
ST_002	yes	missense	c.423G>C	p.C141W	DNA-binding domain	76.04	55.11
ST_007	yes	mssense	c.707T>C	p.Y236C	DNA-binding domain	49.59	36.27
ST_009	yes	missense	c.659T>C	p.Y220C	DNA-binding domain	72.30	50.00
ST_011	yes	missense	c.818C>T	p.R273H	DNA-binding domain	92.86	64.00
ST_013	yes	frame shift deletion	C.636A>-	p.R213fs	DNA-binding domain	92.54	78.82
ST_014	yes	missense	c.818C>T	p.R273H	DNA-binding domain	47.57	25.00
ST_015	yes	frame shift deletion	c.250del	p.A84fs	NA	38.18	29.93
ST_015	no	5' UTR	c.-7579816G>T	NULL	NA	6.59	NA
ST_016	yes	frame shift deletion	c.297_294GGAA>-	p.S99fs	DNA-binding domain	65.69	39.93
ST_017	yes	missense	c.487A>T	p.Y163N	DNA-binding domain	53.65	77.71
ST_018	yes	frame shift insertion	c.371_370->A	p.C124fs	DNA-binding domain	53.23	50.00
ST_019	yes	in-frame deletion	c.478_473TGGCGC >-	p.RAM158in_f rame_delL	DNA-binding domain	NA	NA
ST_020	yes	missense	c.830C>A	p.C277F	DNA-binding domain	21.28	72.41
ST_021	yes	splice site	c.560-1C>T	e5-1	NA	30.63	25.90
ST_023	yes	nonsense	c.499G>A	p.Q167*	DNA-binding domain	18.60	50.00
ST_025	yes	missense	c.490T>C	p.K164E	DNA-binding domain	49.77	62.31
ST_026	yes	missense	c.580G>A	p.L194F	DNA-binding domain	53.37	55.75
ST_027	yes	frame shift deletion	c.1024G>-	p.R342fs	tetramer	51.16	54.20
ST_028	no	splice site	c.376-2T>C	e4-2	NA	95.05	NA
ST_029	yes	missense	c.764A>C	p.I255S	DNA-binding domain	76.10	89.09
ST_030	yes	missense	c.817G>A	p.R273C	DNA-binding domain	76.00	22.22
LT_031	yes	missense	c.722G>A	p.S241F	DNA-binding domain	63.24	46.01
LT_033	yes	missense	c.524C>T	p.R175H	DNA-binding domain	73.97	67.80
LT_033	yes	intronic	c.96+41_96+34 CTCCAGGT>-	e2+41	NA	NA	NA
LT_034	yes	missense	c.818C>T	p.R273H	DNA-binding domain	23.85	15.97
LT_035	yes	splice site	c.672+1C>1	e5+1	NA	57.79	45.45
LT_036	yes	missense	c.584A>G	p.I195T	DNA-binding domain	90.16	81.64
LT_037	yes	nonsense	c.493G>A	p.Q165*	DNA-binding domain	67.61	80.99

LT_038	yes	missense	c.743C>T	p.R248Q	DNA-binding domain	83.19	61.97
LT_039	yes	nonsense	c.592C>A	p.E198*	DNA-binding domain	70.54	41.90
LT_041	yes	missense	c.748G>A	p.P250S	DNA-binding domain	62.39	53.60
LT_041	yes	missense	c.747C>A	p.R249S	DNA-binding domain	62.39	53.28
LT_041	no	intronic	c.96+41_96+3CTCC AGGT>-	e2+41	NA	NA	NA
LT_042	yes	frame shift deletion	c.366_350 CACAGACTTGGC TGTCC>1	p.G117fs	DNA-binding domain	NA	NA
LT_043	yes	frame shift deletion	c.801C>-	p.N268fs	DNA-binding domain	82.26	83.78
LT_043	yes	intronic	c.96+41_96+34 CTCCAGGT>-	e2+41	NA	NA	NA
LT_044	yes	missense	c.488T>G	p.Y163S	DNA-binding domain	41.98	96.08
LT_045	yes	missense	c.638C>A	p.R213L	DNA-binding domain	81.82	77.00
LT_046	yes	frame shift insertion	c.853_852->T	p.E284fs	DNA-binding domain	66.67	17.59

Somatic TP53 mutations identified in tumor cohort from WES.

Supplementary Table 6 INTEGRATE Recurrent Gene Fusions

Recurrent gene fusions unique to ST survivors	Number of samples	Number of patients	Number of primary samples	Number of met samples
LINC01176>>ZNRF2	11	10	4	7
ZRANB1>>AL731577.2	10	9	4	6
CCDC32>>CBX3	9	9	6	3
AC060766.7>>SLFN12	9	9	5	4
AL035409.1>>ST6GALNAC5	9	8	5	4
TIAM2>>SCAF8	10	8	6	4
SLC35A1>>AL109930.1/AL354993.1	7	6	3	4
SLC35A1>>AL354993.1	6	5	2	4
AC025178.1>>GOLPH3	5	5	3	2
BMPR1B>>PDLIM5	4	4	3	1
AL049697.1/SLC35A1>>AL109930.1/AL354993.1	3	3	2	1
SLC35A1>>AL109930.1	4	3	1	3
BACH1-IT1>>BACH1	3	3	2	1
FP236383.1>>PDE3A	3	3	2	1
HYDIN>>AC239809.3	3	3	2	1
AL596220.1>>C1orf27	3	3	1	2
PPP1CB>>PLB1	3	2	2	1
COL14A1>>DEPTOR	3	2	1	2
CCDC6>>ANK3	2	2	1	1
SLC35A1>>AL354993.1/AL109930.1	2	2	1	1
TRIO>>ARHGEF3	3	2	2	1
FP236383.1>>COL6A1	2	2	0	2
RNU6-4P>>SNORA20/TCP1	2	2	1	1
ERBB4>>IKZF2	2	2	1	1
TPTE2>>MRPS31P2	2	2	2	0
CATSPERB>>TC2N	2	2	2	0
PSPC1>>ZMYM5	3	2	1	2
RNU6-2>>SNORD10	2	2	2	0
COL4A2>>COL4A1	2	2	1	1
NCALD>>GRHL2	2	2	2	0
LINC00032>>EQTN	2	2	1	1
MECOM>>PHC3	2	2	2	0
RAD9A>>KDM2A	2	2	2	0
SKIL>>PRKCI	2	2	1	1
CYP1B1-AS1>>RMDN2	2	2	2	0

Chart showing the recurrent gene fusions predicted by INTEGRATE for the ST survivor tumor samples. The chart displays a breakdown of number and types of samples each gene fusion was identified among the ST survivors.

Supplementary Table 7 Predicted Collagen Gene Fusions

Gene fusion (5'>>3')	Patient sample	Encompassing and spanning reads detected in sample	In-Frame prediction
COL1A1>>7SK/RN7SK	ST.015.M.FF	8	out-of-frame
COL1A1>>GSN	ST.001.M.FF	4	in-frame
BROX>>COL1A1	ST.015.M.FF	2	in-frame
MALAT1>>COL1A2	ST.018.M.FFPE	76	in-frame
COL1A2>>NEAT1	ST.029.M.FFPE	40	out-of-frame
C3>>COL1A2	ST.027.P.FF	20	in-frame
RPS11>>COL1A2	ST.018.P.FF	9	in-frame
COL1A2>>PABPC1	ST.001.M.FF	7	in-frame
COL1A2>>PCBP2/AC023509.1	ST.002.M.FF	4	in-frame
AC093752.1>>COL1A2	ST.001.M.FF	3	in-frame
COL1A2>>TBC1D4	ST.018.P.FF	3	out-of-frame
COL1A2>>PCBP2	ST.002.M.FF	2	in-frame
COL1A2>>TBCCD1	ST.001.M.FF	2	in-frame
COL1A2>>YWHAB	ST.029.P.FF	2	in-frame
HM13>>COL1A2	ST.021.P.FF	2	in-frame
MALAT1>>COL3A1	ST.028.M.FFPE	169	in-frame
FN1>>COL3A1	ST.018.P.FF	54	in-frame
COL3A1>>RN7SL2	ST.001.M.FF	52	out-of-frame
COL3A1>>RMRP	ST.021.P.FF	10	out-of-frame
CLU>>COL3A1	ST.029.P.FF	4	in-frame
STX7>>COL3A1	ST.029.P.FF	3	out-of-frame
HAX1>>COL3A1	ST.018.P.FF	3	in-frame
MAP2>>COL3A1	ST.018.P.FF	3	in-frame
COL4A2>>COL4A1	ST.001.M.FF<br>ST.011.P.FF	5 2	in-frame<br>in-frame
RPL15>>COL4A2	ST.030.M.FF	2	out-of-frame
FP236383.1>>COL6A1	ST.023.M.FFPE<br>ST.028.M.FFPE	15<br>28	out-of-frame
COL6A3>>ARID1B	ST.021.P.FF	3	out-of-frame
COL6A2>>FP236383.3	LT.041.M.FFPE	7	out-of-frame
ORC4>>COL6A3	ST.011.M.FF	2	out-of-frame
COL6A6>>TTLL9	ST.027.P.FF	6	out-of-frame
RPS24>>COL12A1	ST.018.P.FF	4	out-of-frame

COL14A1>>DEPTOR	ST.007.P.FF<br>ST.007.M.FF<br>ST.030.M.FF	49<br>3<br>39	in-frame
TFDP2>>COL18A1	ST.013.M.FF	3	in-frame

Chart displaying the 33 gene fusions involving collagen genes, the sample the fusion was identified in, the number of encompassing and spanning reads that identified the fusion, and INTEGRATE's prediction if the reads were in- or out-of-frame.

Supplementary Table 8: CIBERSORT immune cell fraction statistics

	ST v LT survivors		Primary (P) v Metastatic (M)		ST survivors - PvM		LT survivors - PvM		Primary tumors - ST v LT		Metastatic tumors - ST v LT	
Immune cell type	Mann-Whitney Statistic	p-value	Wilcoxon Statistic	p-value	Wilcoxon Statistic	p-value	Wilcoxon Statistic	p-value	Mann-Whitney Statistic	p-value	Mann-Whitney Statistic	p-value
B cells naive	690	0.28222397	51	0.379374669	32	0.58291955	3	0.46520882	177	0.37074399	167	0.593786144
B cells memory	647	0.72285701	196	0.203540906	82	0.60087101	24	0.13295729	164	0.75343757	158	0.909458667
Plasma cells	696.5	0.24182612	52	0.408044206	32	0.58291955	4	0.71500065	189.5	0.11864898	157	0.922974403
T cells CD8	733	0.17680624	187	0.036035175	49	0.01032448	45	0.97212533	154.5	1	217.5	0.040789302
T cells CD4 naive	784	0.00287582	23	0.646462203	23	0.6464622	NA	NA	189	0.06265679	203	0.022749719
T cells CD4 memory resting	690	0.3958775	312	0.741458844	104	0.48259115	24	0.07849121	208	0.08255079	133	0.505906885
T cells CD4 memory activated	658	0.37963095	2	0.025061844	1	0.04639946	0	0.17971249	161	0.45884883	169	0.495912321
T cells follicular helper	356	0.00271976	259	0.359021078	100	0.5900638	34	0.26757813	84	0.02411617	93	0.049561507
T cells regulatory (Tregs)	375	0.00298062	144	0.279641534	43	0.86130426	12	0.00854492	53	0.0004148	145	0.770623805
T cells gamma delta	658	0.16619833	1	0.285049407	1	0.28504941	NA	NA	161	0.45884883	168	0.269900195
NK cells resting	581	0.65898278	141	0.797068494	26	0.17295492	4	0.00992549	205.5	0.05963849	80	0.009823086
NK cells activated	490.5	0.1478352	259	0.925510561	75	0.42090992	27	0.19605096	105	0.11421541	149	0.883753087
Monocytes	676	0.49160656	301	0.615148209	119	0.82372856	40	0.46313477	168	0.66061466	164	0.757858154
Macrophages M0	691	0.38808951	202	0.06419212	67	0.05424547	39	0.64964403	181	0.38921972	163.5	0.769031965
Macrophages M1	505	0.19618414	223	0.304232069	68	0.27724064	48	0.80773926	109	0.13385961	150.5	0.9222738
Macrophages M2	499	0.17838381	321	0.850463631	115	0.72618389	41	0.50158691	134	0.52685427	115	0.211546541
Dendritic cells resting	664	0.41330065	34	0.694886602	15	0.67442407	5	1	162	0.70775726	170	0.466676921
Dendritic cells activated	452	0.05303165	227	0.909930768	72	0.55657941	29	0.43276758	134	0.51125239	93	0.0464126
Mast cells resting	606	0.90155873	111	0.875710309	25	0.15195619	6	0.09289194	139.5	0.60513707	166	0.676859087
Mast cells activated	625	0.91640813	137	0.211555387	75	0.94339206	6	0.02841686	124.5	0.32455326	183.5	0.301292617
Eosinophils	715	0.24089382	180	0.828812374	85	0.98262729	19	0.67840238	174	0.51689552	182	0.354247644
Neutrophils	587.5	0.71791202	78	0.039672486	21	0.04798975	22	0.57506213	155	0.98413058	143	0.718726623

Table listing the Mann-Whitney or Wilcoxon Statistic and corresponding p-values for comparisons between ST and LT survivors and Primary and Metastatic tumor for each of the 22 immune cell types

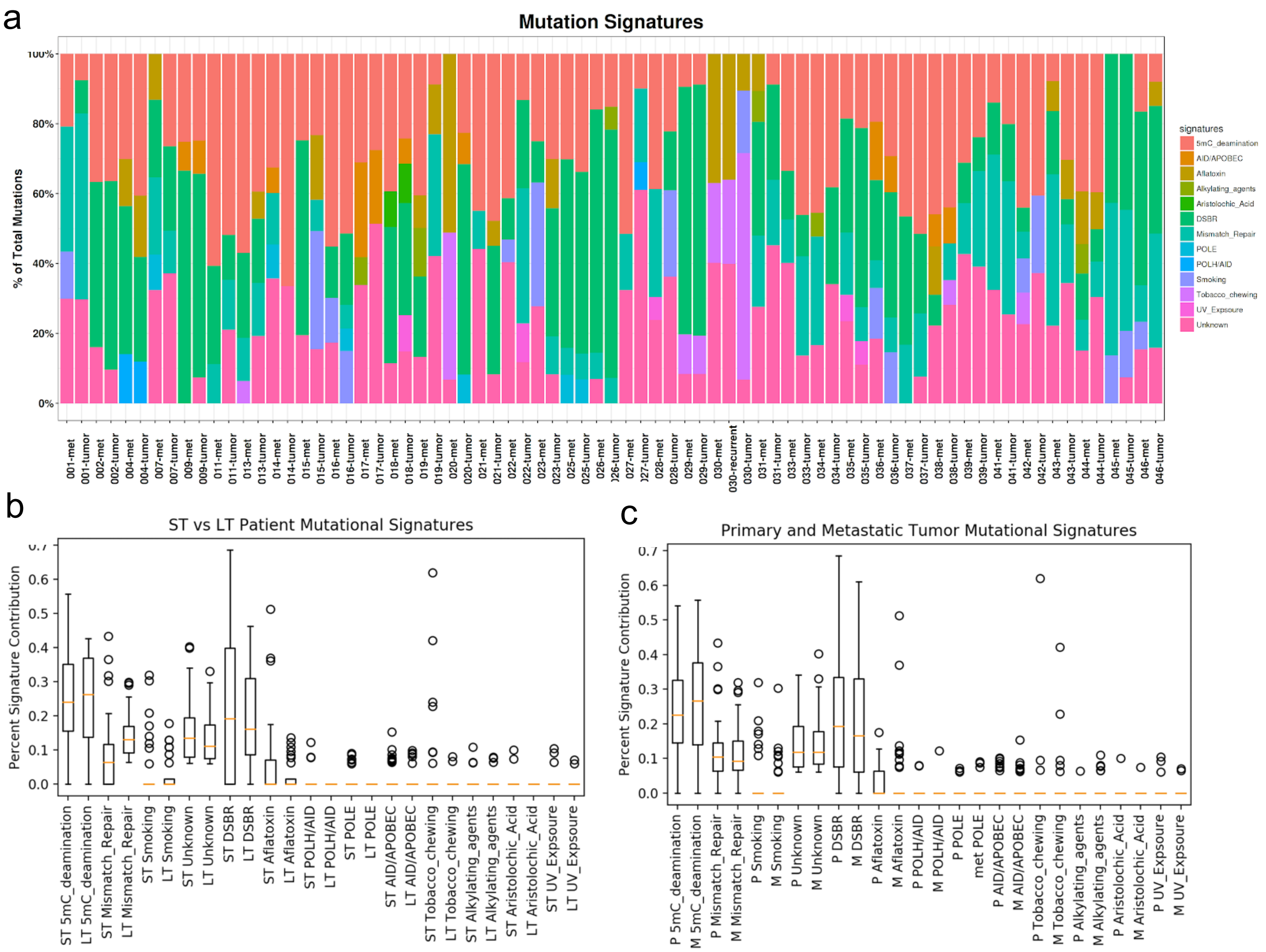

Supplementary Fig 1 Mutational Signatures

a. Stacked bar plot displaying the percent of total mutations that fit into the mutational signatures shown on the right. Each patient is ordered by patient number and their metastatic and primary tumor with ST survivors on the left (001-met – 030 tumor) and LT survivors on the right (031-met – 046-tumor).

b-c. Box and whisker plots showing the percentage of total mutations for each signature, grouped by the patient's tumor as a ST or LT survivor (B) or as a primary (P) and metastatic tumor (M) (C). The boxplots define the range of the minimum to the maximum by lines, a box from the first quartile to the third quartile with the median as the center line. Circles outside the range of the data are outliers.

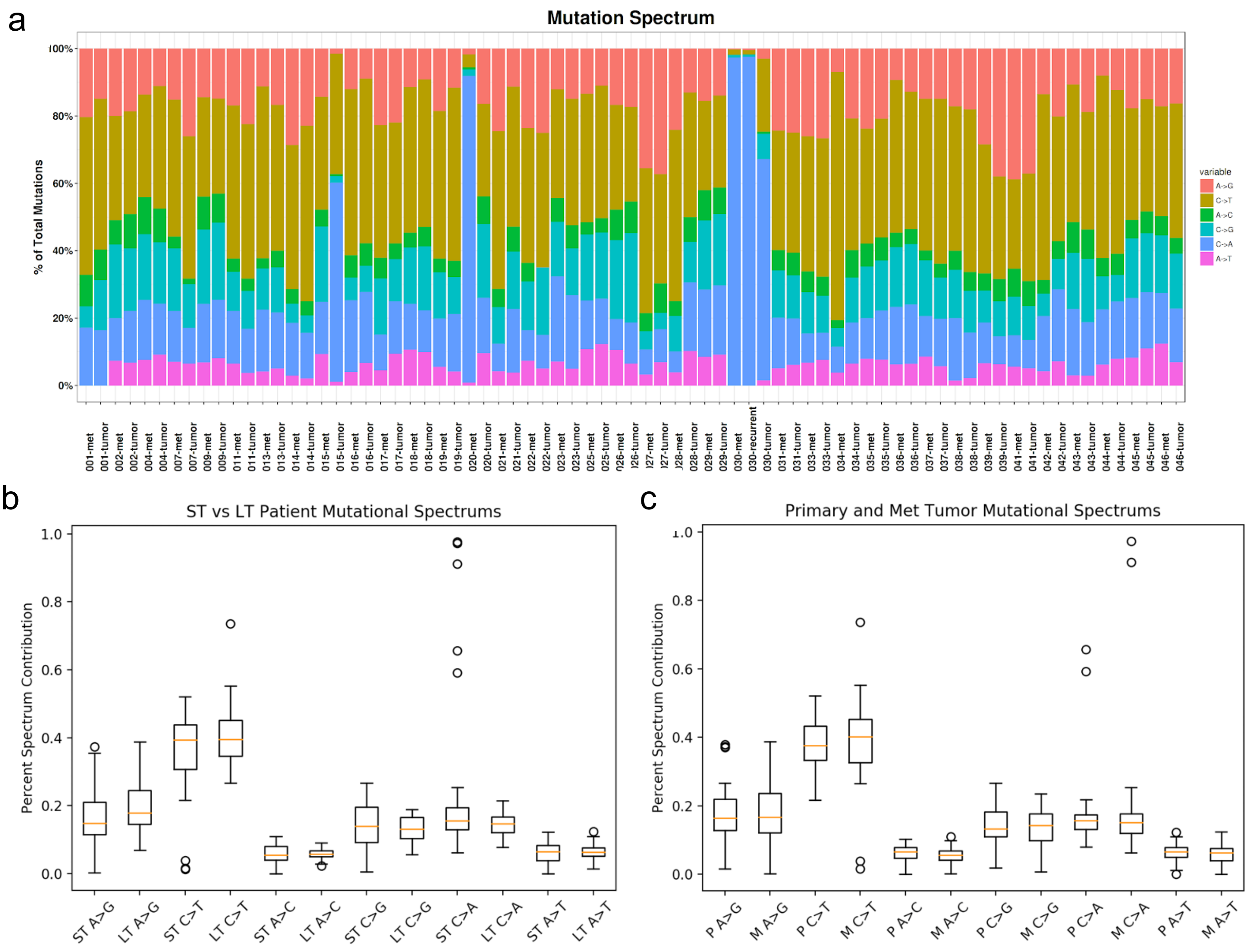

Supplementary Fig 2 Mutational Spectrum

a. Stacked bar plot displaying the percent of total mutations that were nucleotide transitions or transversions, according to the key on the right. Each patient is ordered by patient number and their metastatic and primary tumor with ST survivors on the left (001-met – 030 tumor) and LT survivors on the right (031-met – 046-tumor).

b-c. Box and whisker plots showing the percentage of total mutations for each signature, grouped by the patient's tumor as a ST or LT survivor (B) or as a primary (P) and metastatic tumor (M) (C). The boxplots define the range of the minimum to the maximum by lines, a box from the first quartile to the third quartile with the median as the center line. Circles outside the range of the data are outliers.

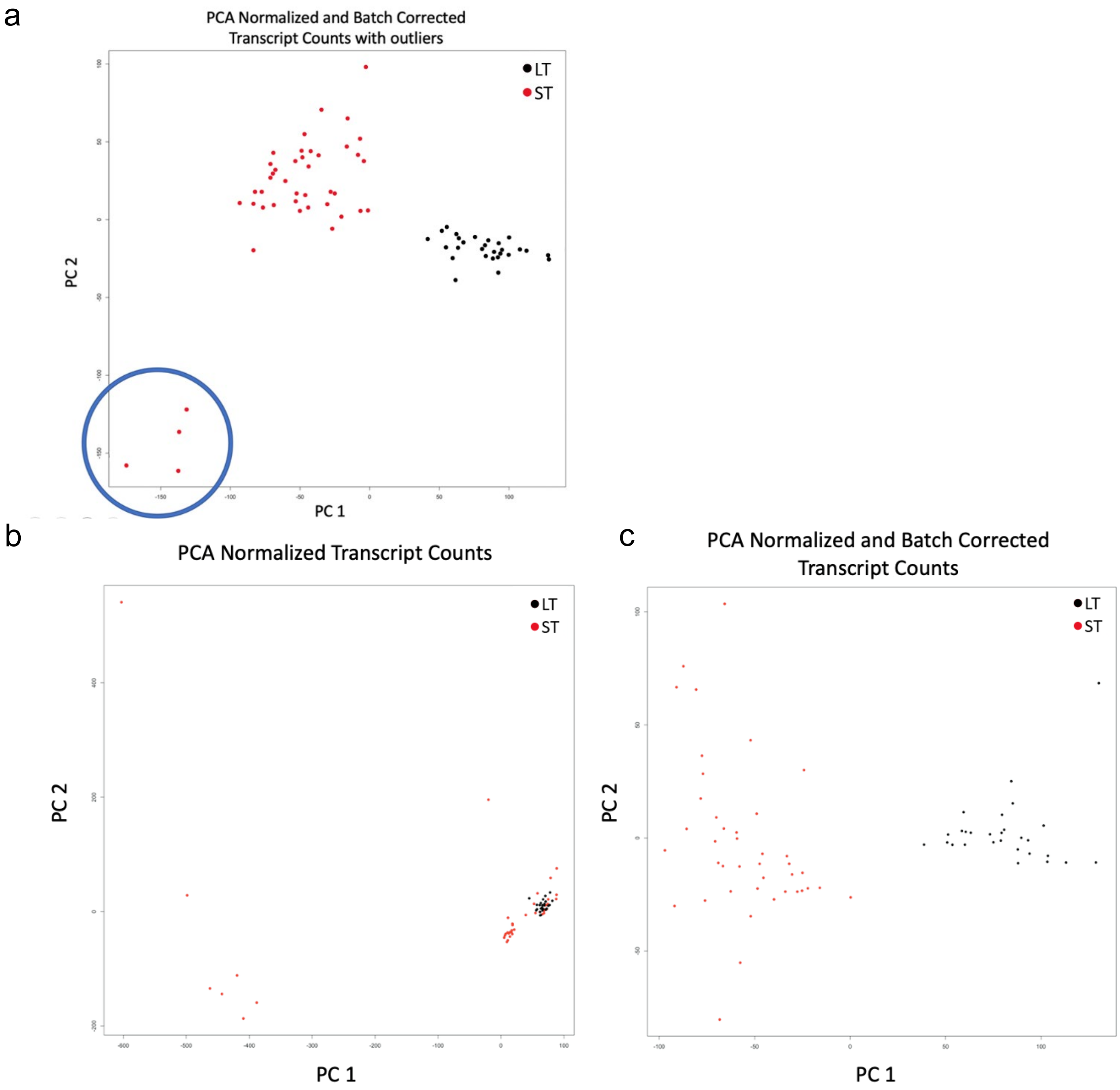

Supplementary Fig 3 PCA Plots of Log CPM counts in Differential Expression Analysis

a. PCA of cohort samples RNA-sequencing transcript counts with all tumor samples after normalization and batch effect correction with SVA. The 4 samples circled in the plot were removed as outliers.
b. PCA of cohort samples RNA-sequencing transcript counts after removal of the 4 outlier samples and normalization.
c. PCA of cohort samples RNA-sequencing transcript counts after removal of the 4 outlier samples, normalization, and batch effect correction with SVA.

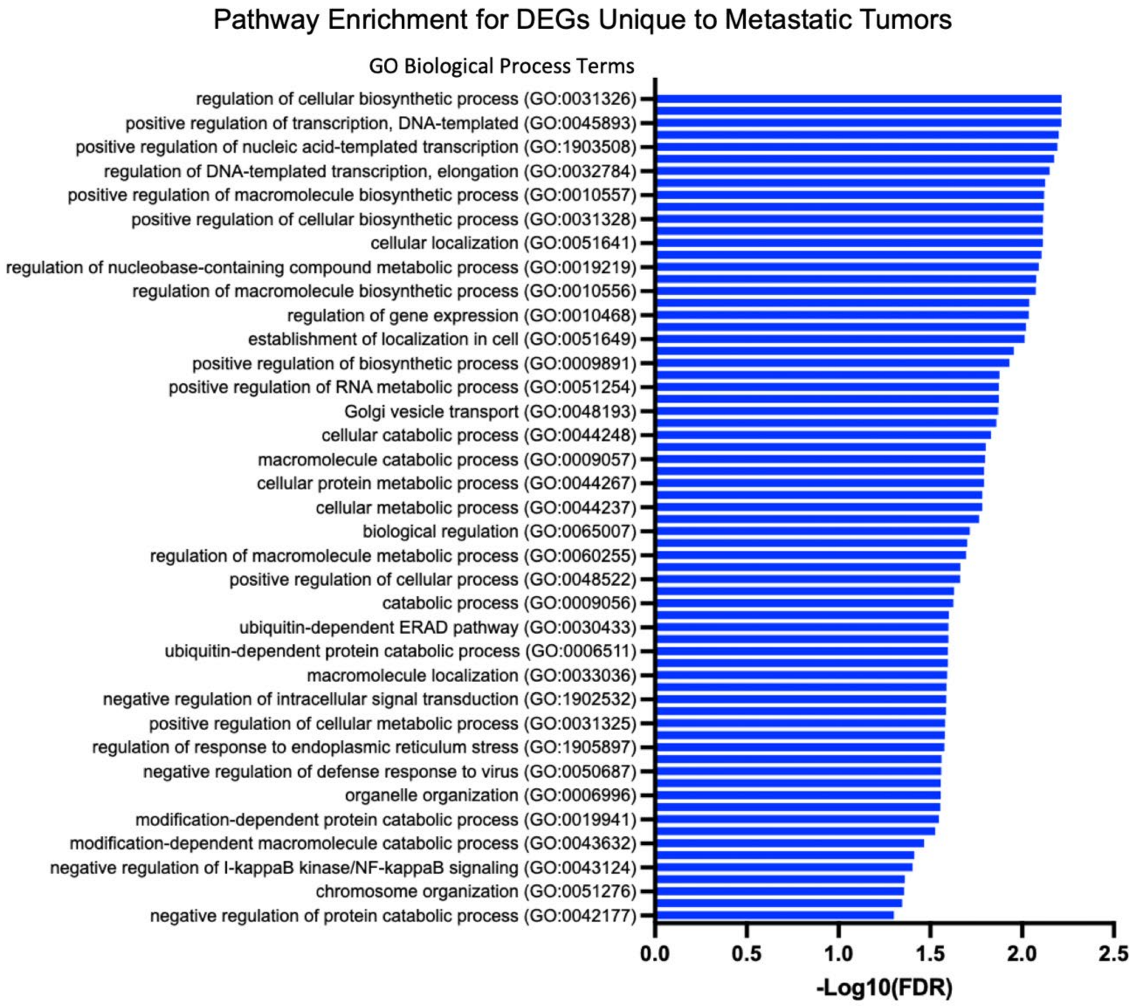

Supplementary Fig 4 GO Biological Process Pathway Enrichment for Metastatic DEGs Barplot displaying the -Log10(FDR) values for each Biological Processes GO term that was statistically overrepresented among the DEGs unique to metastatic tumors

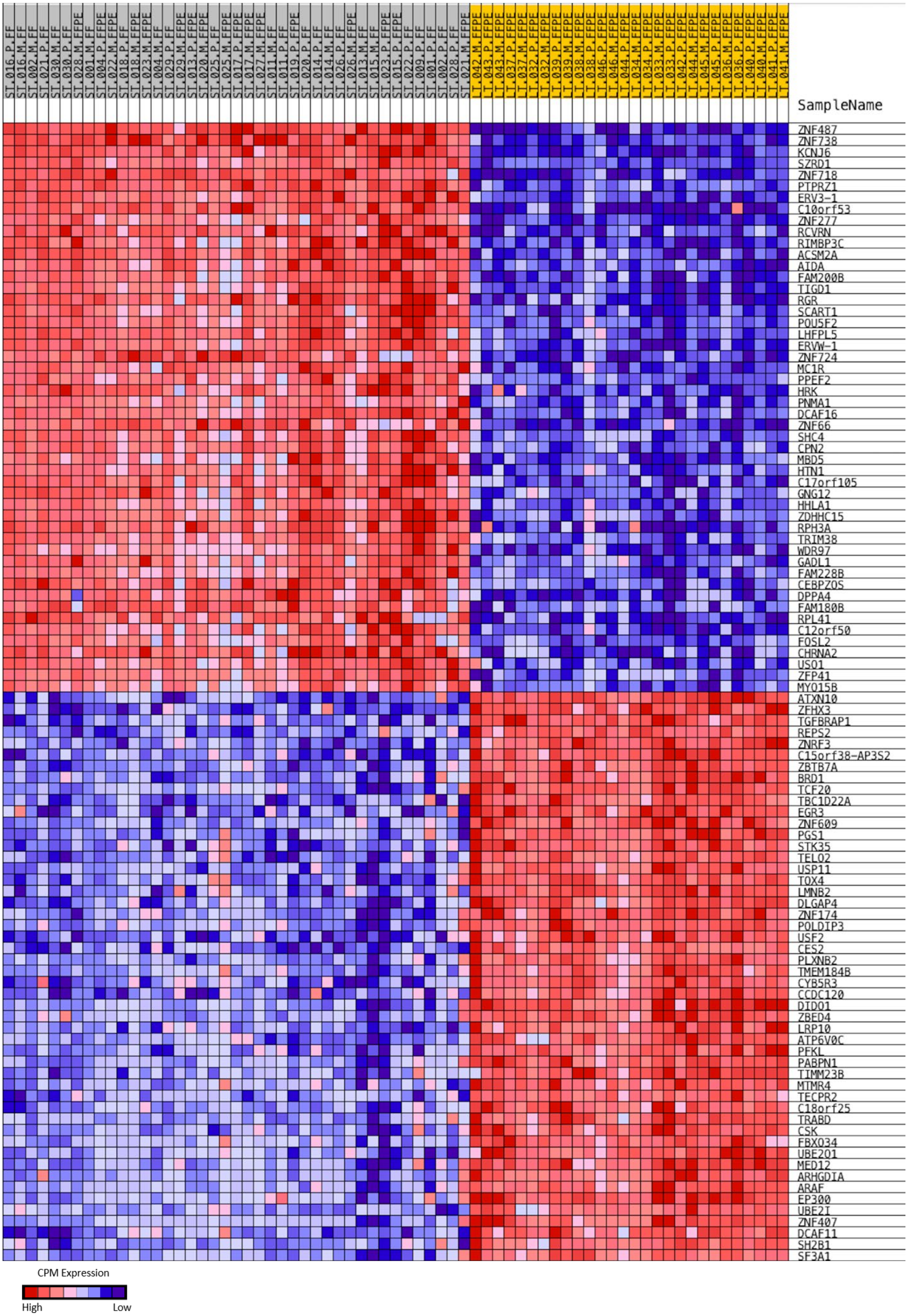
ST.016.P.FF
ST.016.M.FF
ST.002.M.FF
ST.021.P.FF
ST.030.M.FF
ST.030.P.FF
ST.028.M.FFPE
ST.001.M.FF
ST.004.P.FFPE
ST.022.M.FFPE
ST.018.P.FF
ST.018.M.FFPE
ST.023.M.FFPE
ST.004.M.FF
ST.029.P.FF
ST.029.M.FFPE
ST.013.P.FFPE
ST.020.M.FFPE
ST.025.P.FF
ST.025.M.FFPE
ST.017.P.FFPE
ST.017.M.FFPE
ST.027.M.FFPE
ST.011.M.FF
ST.011.P.FF
ST.019.P.FFPE
ST.020.P.FF
ST.014.P.FF
ST.014.M.FF
ST.026.P.FF
ST.026.M.FFPE
ST.013.M.FF
ST.015.M.FF
ST.023.P.FFPE
ST.015.P.FFPE
ST.022.P.FF
ST.009.P.FF
ST.001.P.FF
ST.002.P.FF
ST.028.P.FF
ST.021.M.FFPE
LT.042.M.FFPE
LT.043.P.FFPE
LT.043.M.FFPE
LT.037.P.FFPE
LT.037.M.FFPE
LT.032.P.FFPE
LT.032.M.FFPE
LT.039.P.FFPE
LT.039.M.FFPE
LT.038.M.FFPE
LT.038.P.FFPE
LT.046.P.FFPE
LT.046.M.FFPE
LT.044.P.FFPE
LT.034.M.FFPE
LT.034.P.FFPE
LT.033.M.FFPE
LT.033.P.FFPE
LT.042.P.FFPE
LT.044.M.FFPE
LT.045.M.FFPE
LT.045.P.FFPE
LT.036.M.FFPE
LT.036.P.FFPE
LT.040.P.FFPE
LT.040.M.FFPE
LT.041.P.FFPE
LT.041.M.FFPE
SampleName
ZNF487
ZNF738
KCNJ6
SZRD1
ZNF718
PTPRZ1
ERV3-1
C10orf53
ZNF277
RCVRN
RIMBP3C
ACSM2A
AIDA
FAM200B
TIGD1
RGR
SCART1
POU5F2
LHFPL5
ERVW-1
ZNF724
MC1R
PPEF2
HRK
PNMA1
DCAF16
ZNF66
SHC4
CPN2
MBD5
HTN1
C17orf105
GNG12
HHLA1
ZDHHC15
RPH3A
TRIM38
WDR97
GADL1
FAM228B
CEBPZOS
DPPA4
FAM180B
RPL41
C12orf50
FOSL2
CHRNA2
USO1
ZFP41
MYO15B
ATXN10
ZFHX3
TGFBRAP1
REPS2
ZNRF3
C15orf38-AP3S2
ZBTB7A
BRD1
TCF20
TBC1D22A
EGR3
ZNF609
PGS1
STK35
TELO2
USP11
TOX4
LMNB2
DLGAP4
ZNF174
POLDIP3
USF2
CES2
PLXNB2
TMEM184B
CYB5R3
CCDC120
DIDO1
ZBED4
LRP10
ATP6V0C
PFKL
PABPN1
TIMM23B
MTMR4
TECPR2
C18orf25
TRABD
CSK
FBXO34
UBE2O1
MED12
ARHGDIA
ARAF
EP300
UBE2I
ZNF407
DCAF11
SH2B1
SF3A1
CPM Expression
High
Low

Supplementary Fig 5 Top upregulated and downregulated DEGs between ST and LT survivors Heatmap displaying the top 50 upregulated and top 50 downregulated DEGs between the all ST and LT survivors generated by GSEA.

Category	Term	RT	Genes	Count	%	P-Value	Fold Enrichment	Benjamini	FDR	Fisher Exact
KEGG_PATHWAY	Adherens junction	RT		6	1.9	5.2E-3	5.3	6.2E-1	6.2E-1	8.9E-4
KEGG_PATHWAY	Protein processing in endoplasmic reticulum	RT		9	2.8	5.6E-3	3.3	6.2E-1	6.2E-1	1.6E-3
KEGG_PATHWAY	SNARE interactions in vesicular transport	RT		4	1.3	1.5E-2	7.6	1.0E0	1.0E0	1.8E-3
KEGG_PATHWAY	Alcoholic liver disease	RT		7	2.2	2.5E-2	3.1	1.0E0	1.0E0	7.4E-3
KEGG_PATHWAY	mTOR signaling pathway	RT		7	2.2	3.7E-2	2.8	1.0E0	1.0E0	1.2E-2
KEGG_PATHWAY	mRNA surveillance pathway	RT		5	1.6	6.7E-2	3.2	1.0E0	1.0E0	1.9E-2
KEGG_PATHWAY	Phospholipase D signaling pathway	RT		6	1.9	8.5E-2	2.5	1.0E0	1.0E0	3.1E-2
KEGG_PATHWAY	Insulin resistance	RT		5	1.6	9.1E-2	2.9	1.0E0	1.0E0	2.9E-2
KEGG_PATHWAY	MAPK signaling pathway	RT		9	2.8	9.4E-2	1.9	1.0E0	1.0E0	4.5E-2

Supplementary Fig 6 Kegg Pathway Enrichment
KEGG Pathways enriched in DEGs as identified by DAVID

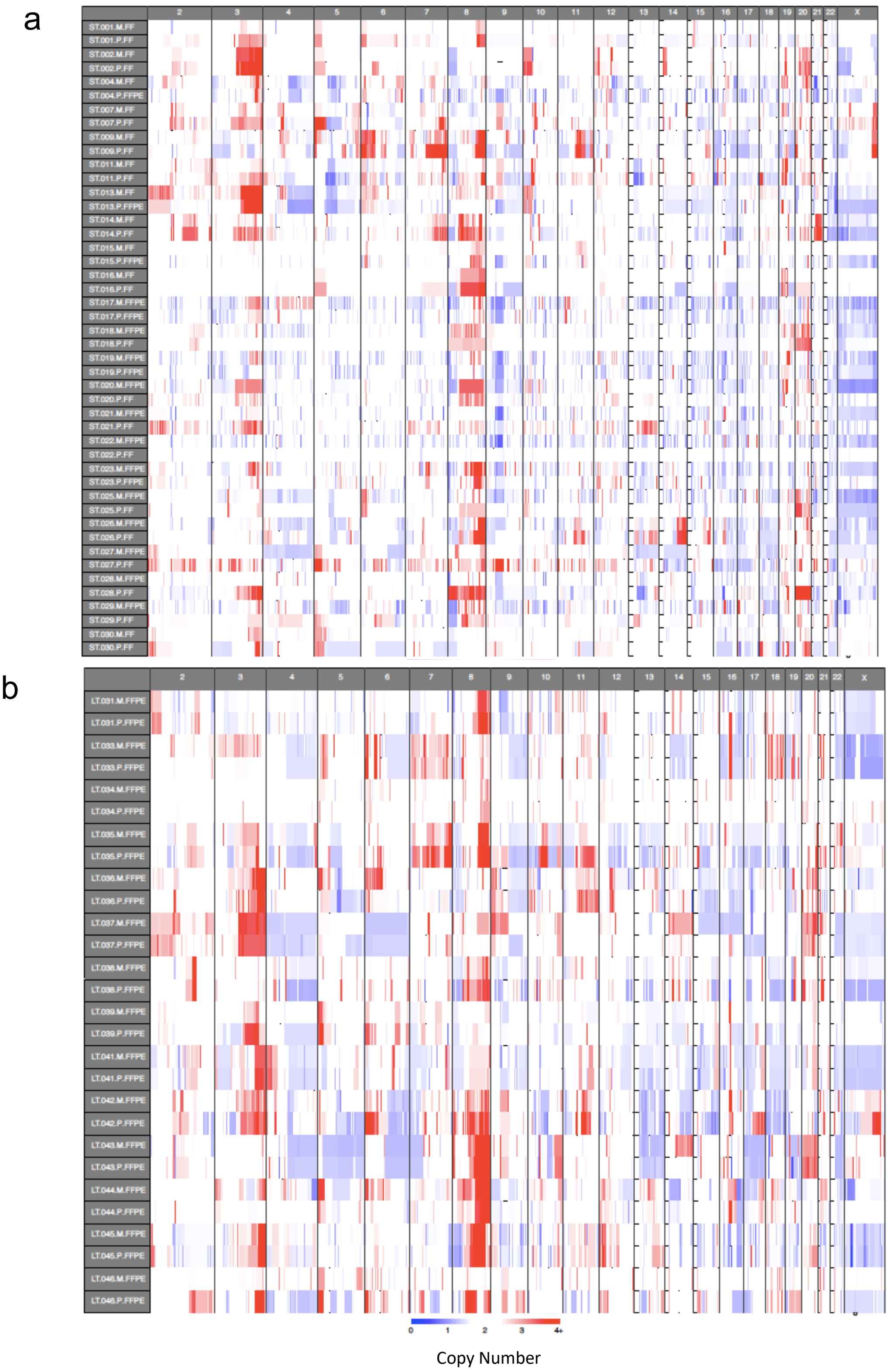
a
b
2 3 4 5 6 7 8 9 10 11 12 13 14 15 16 17 18 19 20 21 22 X
ST.001.M.FF
ST.001.P.FF
ST.002.M.FF
ST.002.P.FF
ST.004.M.FF
ST.004.P.FFPE
ST.007.M.FF
ST.007.P.FF
ST.009.M.FF
ST.009.P.FF
ST.011.M.FF
ST.011.P.FF
ST.013.M.FF
ST.013.P.FFPE
ST.014.M.FF
ST.014.P.FF
ST.015.M.FF
ST.015.P.FFPE
ST.016.M.FF
ST.016.P.FF
ST.017.M.FFPE
ST.017.P.FFPE
ST.018.M.FFPE
ST.018.P.FF
ST.019.M.FFPE
ST.019.P.FFPE
ST.020.M.FFPE
ST.020.P.FF
ST.021.M.FFPE
ST.021.P.FF
ST.022.M.FFPE
ST.022.P.FF
ST.023.M.FFPE
ST.023.P.FFPE
ST.025.M.FFPE
ST.025.P.FF
ST.026.M.FFPE
ST.026.P.FF
ST.027.M.FFPE
ST.027.P.FF
ST.028.M.FFPE
ST.028.P.FF
ST.029.M.FFPE
ST.029.P.FF
ST.030.M.FF
ST.030.P.FF
LT.031.M.FFPE
LT.031.P.FFPE
LT.033.M.FFPE
LT.033.P.FFPE
LT.034.M.FFPE
LT.034.P.FFPE
LT.035.M.FFPE
LT.035.P.FFPE
LT.036.M.FFPE
LT.036.P.FFPE
LT.037.M.FFPE
LT.037.P.FFPE
LT.038.M.FFPE
LT.038.P.FFPE
LT.039.M.FFPE
LT.039.P.FFPE
LT.041.M.FFPE
LT.041.P.FFPE
LT.042.M.FFPE
LT.042.P.FFPE
LT.043.M.FFPE
LT.043.P.FFPE
LT.044.M.FFPE
LT.044.P.FFPE
LT.045.M.FFPE
LT.045.P.FFPE
LT.046.M.FFPE
LT.046.P.FFPE
0 1 2 3 4+
Copy Number

Supplementary Fig 7 Copy number alterations in tumors of ST and LT survivors

a. Copy-altered segments in primary and metastatic tumors of ST survivors across the genome. Amplifications are depicted in red while deletions are seen in blue.

b. Copy-altered segments in primary and metastatic tumors of LT survivors across the genome. Amplifications are depicted in red while deletions are seen in blue.

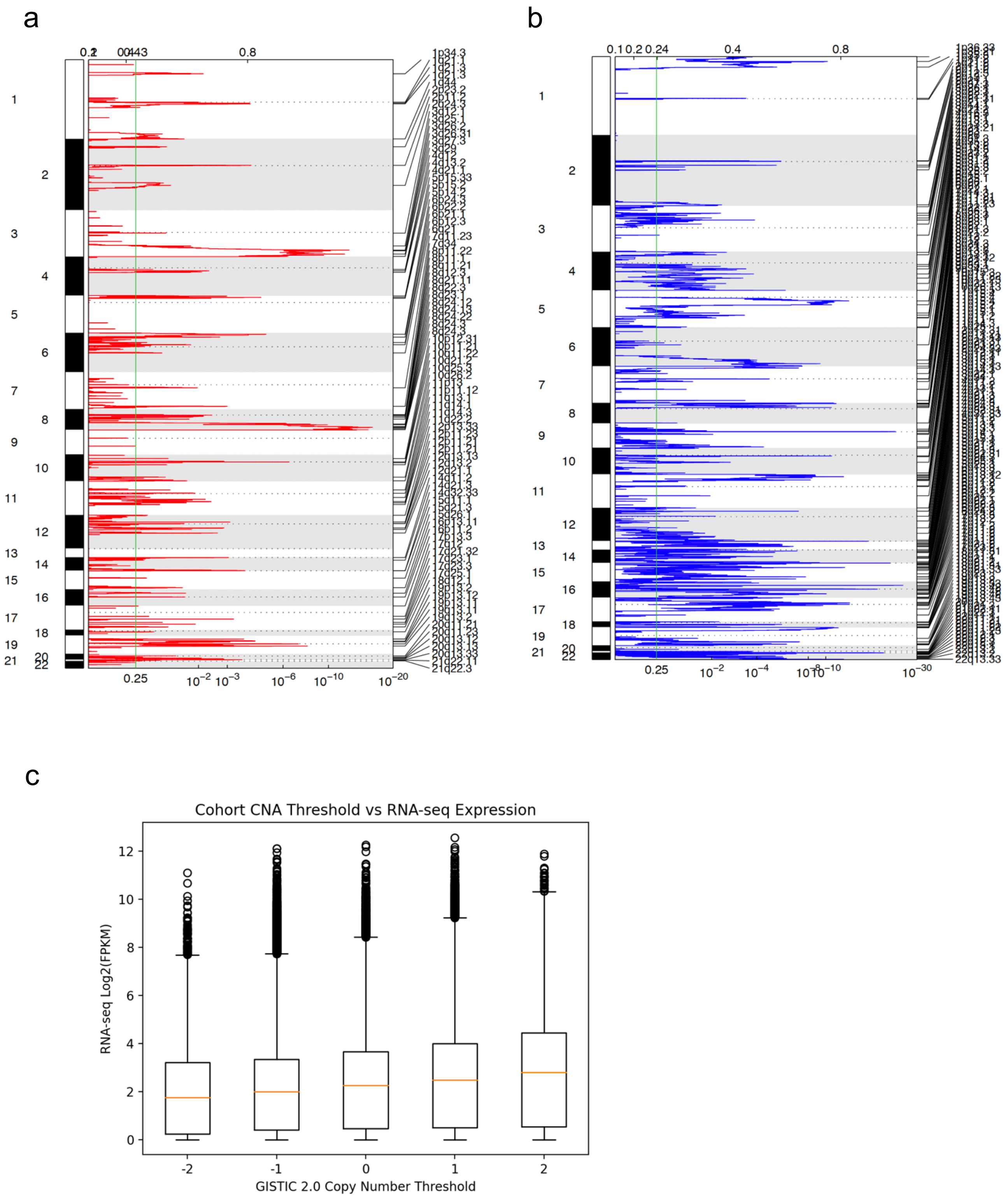
a
b
c
Cohort CNA Threshold vs RNA-seq Expression
RNA-seq Log2(FPKM)
GISTIC 2.0 Copy Number Threshold

Supplementary Fig 8 GISTIC analysis for copy number alterations in tumors of ST and LT survivors

a. GISTIC Qplot of significantly amplified lesions across genome of all patient tumors (n=74) in cohort with 90% Confidence.

b. GISTIC Qplot of significantly deleted lesions across genome of all patient tumors (n=74) in cohort with 90% Confidence.

c. Boxplot of the GISTIC2.0 CNA value for every gene in every patient primary and metastatic sample plotted against their corresponding Log2(FPKM) value from RNA-seq. The medians of the FPKM values and the CNA threshold values has a spearmanr correlation value of 0.999 and p-value of 1.4x10^-24). (Number of genes in Copy Number Threshold groups from left to right: -2 n= 24316, -1 n= 416616, 0 n=280688, 1 n=346144 , 2 n=51856) The boxplot defines the range of the minimum to the maximum by lines, a box from the first quartile to the third quartile with the median as the center line. Circles outside the range of the data are outliers.

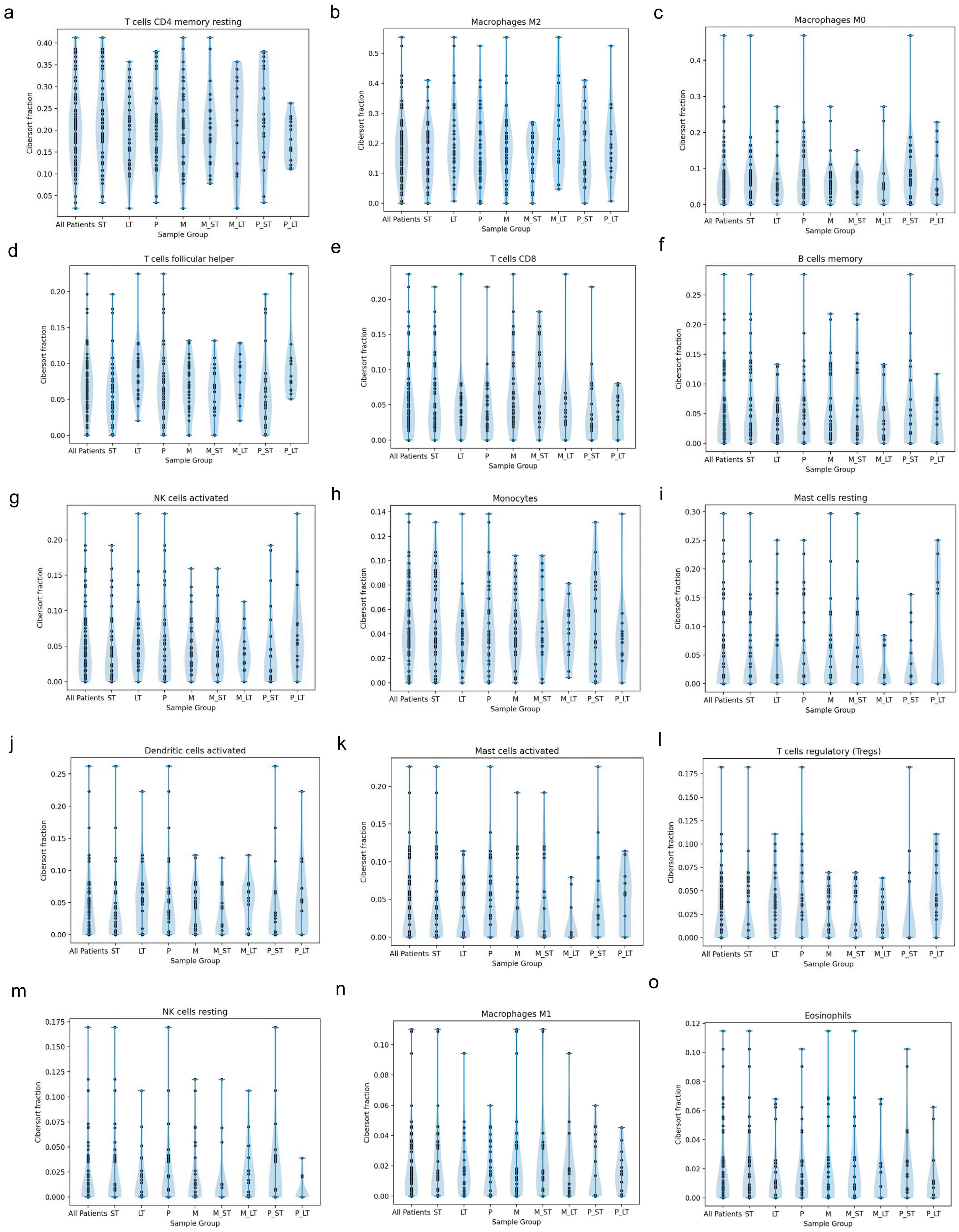
a
T cells CD4 memory resting
Cibersort fraction
All Patients ST LT P M M_ST M_LT P_ST P_LT
Sample Group
b
Macrophages M2
Cibersort fraction
All Patients ST LT P M M_ST M_LT P_ST P_LT
Sample Group
c
Macrophages M0
Cibersort fraction
All Patients ST LT P M M_ST M_LT P_ST P_LT
Sample Group
d
T cells follicular helper
Cibersort fraction
All Patients ST LT P M M_ST M_LT P_ST P_LT
Sample Group
e
T cells CD8
Cibersort fraction
All Patients ST LT P M M_ST M_LT P_ST P_LT
Sample Group
f
B cells memory
Cibersort fraction
All Patients ST LT P M M_ST M_LT P_ST P_LT
Sample Group
g
NK cells activated
Cibersort fraction
All Patients ST LT P M M_ST M_LT P_ST P_LT
Sample Group
h
Monocytes
Cibersort fraction
All Patients ST LT P M M_ST M_LT P_ST P_LT
Sample Group
i
Mast cells resting
Cibersort fraction
All Patients ST LT P M M_ST M_LT P_ST P_LT
Sample Group
j
Dendritic cells activated
Cibersort fraction
All Patients ST LT P M M_ST M_LT P_ST P_LT
Sample Group
k
Mast cells activated
Cibersort fraction
All Patients ST LT P M M_ST M_LT P_ST P_LT
Sample Group
l
T cells regulatory (Tregs)
Cibersort fraction
All Patients ST LT P M M_ST M_LT P_ST P_LT
Sample Group
m
NK cells resting
Cibersort fraction
All Patients ST LT P M M_ST M_LT P_ST P_LT
Sample Group
n
Macrophages M1
Cibersort fraction
All Patients ST LT P M M_ST M_LT P_ST P_LT
Sample Group
o
Eosinophils
Cibersort fraction
All Patients ST LT P M M_ST M_LT P_ST P_LT
Sample Group

p

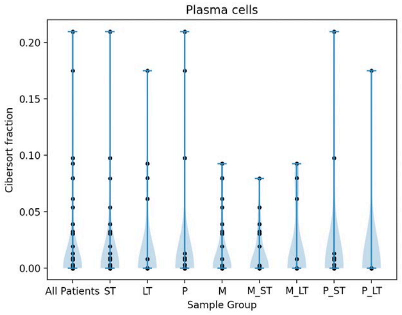

q

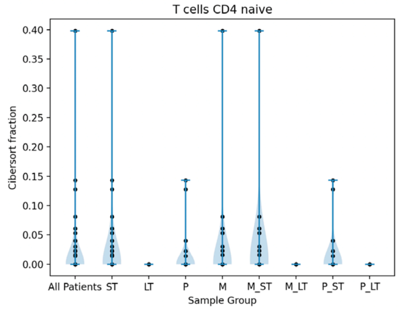

r

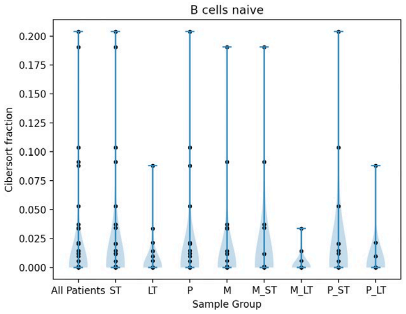

s

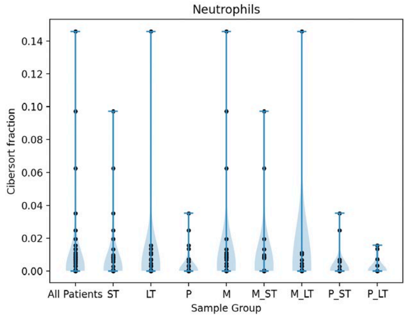

t

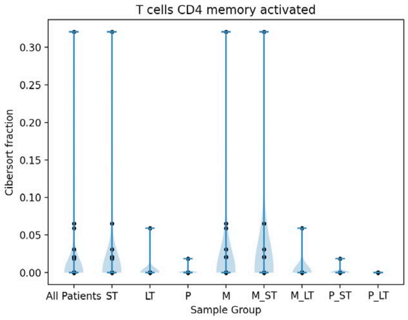

u

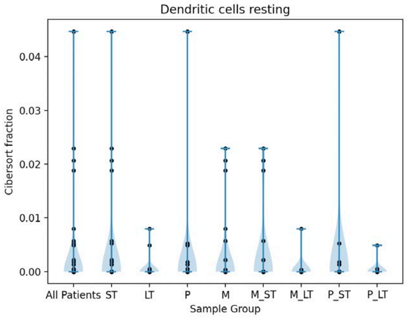

v

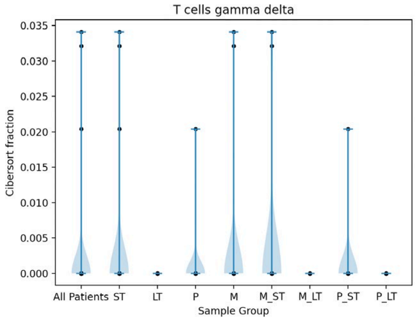

Supplementary Fig 9 CIBERSORT immune cell fraction violin plots

a-v. Breakdown of the immune cell fractions calculated by CIBERSORT for each of the 22 immune cell signatures broken down by tumor type subgroup (all Patient tumors n= 72, ST survivors n= 44, LT survivors n= 28, Primary tumors n = 36, Metastatic tumors n= 36, ST survivor metastatic tumors n= 22, LT survivor metastatic tumors n=14, ST survivor primary tumors n= 22, and LT survivor metastatic tumors n= 14). The bars of the violin plot represent the range of the data (min and max). The shaded region on either side of the center line display the distribution of the data as a kernel density estimation. In other words, it shows the probability that a data point falls within that value range along the y-axis. Each individual data point is shown by a black dot.
