# Supplementary material for: Genetic characterization of primary and metastatic high-grade serous ovarian cancer tumors reveals distinct features associated with survival
Source: Commun Biol. 2023 Jul 3;6:688. doi: 10.1038/s42003-023-05026-3 (PMC10318066; doi:10.1038/s42003-023-05026-3)
Supplement: Supplementary file 3 — Description of Additional Supplementary Data [file 42003_2023_5026_MOESM3_ESM.docx]

**Description of Additional Supplementary Files**

**File name:** Supplementary Data 1

**Description:** Lists of the differentially expressed genes and lncRNAs with their expression values from the heatmaps displayed in the manuscript (each heatmap and dataset is its own tab). One tab lists the predicted gene fusion transcript reads for each patient in the cohort.

**File name:** Supplementary Data 2

**Description:** List of all somatic variants for each tumor sample that was identified by whole exome sequencing. The second tab lists the quality control measures for the RNA-seq bam files.

**File name:** Supplementary Data 3

**Description:** Data sources for Figures 2, 3c, and 4.
