# Supplementary material for: Genetic characterization of primary and metastatic high-grade serous ovarian cancer tumors reveals distinct features associated with survival
Source: Commun Biol. 2023 Jul 3;6:688. doi: 10.1038/s42003-023-05026-3 (PMC10318066; doi:10.1038/s42003-023-05026-3)
Supplement: Supplementary file 7 — Reporting Summary [file 42003_2023_5026_MOESM7_ESM.pdf]

nature portfolio

Corresponding author(s): Dr. Katherine Fuh

Last updated by author(s): 4/27/2023

Reporting Summary

Nature Portfolio wishes to improve the reproducibility of the work that we publish. This form provides structure for consistency and transparency in reporting. For further information on Nature Portfolio policies, see our Editorial Policies and the Editorial Policy Checklist.

Statistics

For all statistical analyses, confirm that the following items are present in the figure legend, table legend, main text, or Methods section.

n/a	Confirmed	
☐	☒	The exact sample size (*n*) for each experimental group/condition, given as a discrete number and unit of measurement
☒	☐	A statement on whether measurements were taken from distinct samples or whether the same sample was measured repeatedly
☐	☒	The statistical test(s) used AND whether they are one- or two-sided<br>*Only common tests should be described solely by name; describe more complex techniques in the Methods section.*
☒	☐	A description of all covariates tested
☒	☐	A description of any assumptions or corrections, such as tests of normality and adjustment for multiple comparisons
☐	☒	A full description of the statistical parameters including central tendency (e.g. means) or other basic estimates (e.g. regression coefficient) AND variation (e.g. standard deviation) or associated estimates of uncertainty (e.g. confidence intervals)
☒	☐	For null hypothesis testing, the test statistic (e.g. *F*, *t*, *r*) with confidence intervals, effect sizes, degrees of freedom and *P* value noted<br>*Give P values as exact values whenever suitable.*
☒	☐	For Bayesian analysis, information on the choice of priors and Markov chain Monte Carlo settings
☐	☒	For hierarchical and complex designs, identification of the appropriate level for tests and full reporting of outcomes
☐	☒	Estimates of effect sizes (e.g. Cohen's *d*, Pearson's *r*), indicating how they were calculated

Our web collection on statistics for biologists contains articles on many of the points above.

Software and code

Policy information about availability of computer code

Data collection

Exome and RNA sequencing
Whole exome sequencing of DNA from matched primary tumor, metastatic tumors, and normal tissue samples was completed for 39 patients with the NimbleGen VCRome exome capture kit (NimbleGen Roche) according to the manufacturer's protocol. Paired-end Illumina 151 bp reads were generated for normal samples. RNA sequencing of primary and metastatic tumor samples was performed using the Illumina TruSeq stranded Total RNA library kit following the Manufacturer-recommended protocol. Paired-end Illumina sequencing of 151 bp read length yielded an average of approximately 125 million paired reads per sample and an average of approximately 134 million reads mapped per sample. Quality Control metrics for the RNA-seq samples were generated using MultiQC and are reported in Supplementary File.

Data analysis

Variant calling and genomic analysis
Exome sequencing data were aligned to human reference build GRCh37 using BWA-mem and deduplicated with Picard version 1.113. Variants were called from the union of 4 callers: Samtools version r932, Somatic Sniper version 1.0.4, VarScan version 2.3.6, Strelka version 1.0.11, and Mutect v1.1. Indels were detected from the union of 4 callers; GATK somatic-indel version 5336, Pindel version 0.5, VarScan version 2.3.6, and Strelka version 1.0.11. SNVs and indels were discarded if they had below 20x coverage, appeared as artifacts in a panel of 905 normal exomes, or exceeded 0.1% frequency in the 1000 genomes or NHLBI exome sequencing projects. A Bayesian classifier (https://github.com/genome/genome/blob/master/lib/perl/Genome/Model/Tools/Validation/IdentifyOutliers.pm). The waterfall plot (Figure 1A-B) depicting frequently mutated genes from TCGA-OV was generated using GenVisR [8, 28]. Mutational clinical significance for somatic and germline BRCA mutations was determined from ClinVar (https://www.ncbi.nlm.nih.gov/clinvar/) (Tables 3-5) [29]. CLOVAR signatures were calculated according to parameters defined in Verhaak et al. [30]. Copy-altered segments were identified from VarScan (Supplementary Figure 5) [21]. Significant copy-altered segments were identified for all tumors, all tumors from using the GISTIC 2.0 version 6.15.28 Module on the AWS GenePattern cloud (https://cloud.genepattern.org/gp).
Differential expression analysis

Transcript read counts were obtained using Kallisto version: v0.43.1 and gene-level read counts were calculated using GRCh37 in Ensembl. Quality control and normalization of the raw count data were performed using the R/Bioconductor package edgeR version 3.28. We used the SVA function of the R/Bioconductor package SVA version 3.34.0 to estimate and remove surrogate variables for unwanted and unknown batch effects and other sources of variation present in the data. DGE analysis was performed using edgeR version 3.28.0, which implements a negative-binomial general linear model. The threshold for significance was set to FDR Q-value < 0.01. We further curated our differentially expressed genes (DEGs) by limiting to protein coding genes that were listed in Ensembl genes 100 Human genes (GRCh38.p13) protein_coding transcript type on BioMart. Pathway analysis was applied to the DEG and gene fusion gene lists using the PANTHER classification system 16.0 (http://pantherdb.org/), with the organisms set as 'Homo sapiens' and performing a statistical overrepresentation test using Fisher's Exact test and calculating a False Discovery Rate. We used all Gene Ontology (GO) terms (Biological Processes, Molecular Function, and Cellular Components), PANTHER pathways, and Reactome pathways annotation sets.
Immune cell abundance estimates
We used Cibersort (https://cibersort.stanford.edu/) to estimate the abundance of infiltrating immune cell types using our tumor RNA-seq data.
Gene fusion predictions
Gene fusion predictions for each tumor sample were produced using INTEGRATE v0.2.6 to analyze the tumor RNA-sequencing data. Full-length raw reads and a set of reads trimmed to remove potentially low-quality bases were each aligned to human reference genome GRCh38 (r90) using STAR v2.5.3a.
Any analyzed data not presented in figures is freely available upon request. Code used to analyze genomic data is publicly available and custom code will be deposited on Github (https://github.com/ekotnik/OC-Tumor-genomic-analyses).

For manuscripts utilizing custom algorithms or software that are central to the research but not yet described in published literature, software must be made available to editors and reviewers. We strongly encourage code deposition in a community repository (e.g. GitHub). See the Nature Portfolio guidelines for submitting code & software for further information.

Data

Policy information about availability of data

All manuscripts must include a data availability statement. This statement should provide the following information, where applicable:

- Accession codes, unique identifiers, or web links for publicly available datasets
- A description of any restrictions on data availability
- For clinical datasets or third party data, please ensure that the statement adheres to our policy

RNA-sequening files have been deposited in the NCBI GEO data base under GSE218939. WES data generated for this analysis have been deposited within the Sequence Read Archive under the accession "PRJNA957243", and can be found at https://www.ncbi.nlm.nih.gov/bioproject/PRJNA957243. Lists of SNVs, DEGs, lncRNAs, and gene fusions are provided in Additional File 1 and 2. Source data for figures have been submitted in Additional File 3.
Code used to analyze genomic data is publicly available and custom code is deposited on Github (https://github.com/ekotnik/OC-Tumor-genomic-analyses) DOI: 10.5281/zenodo.7873762.

Human research participants

Policy information about studies involving human research participants and Sex and Gender in Research.

Reporting on sex and gender	All findings in this manuscript apply to individuals that have ovaries. Our patient cohort consists of only females.
Population characteristics	We collected normal tissue, primary tumor, and metastatic tumor samples from a total of 39 patients diagnosed with FIGO stage III or higher HGSC. Normal tissue samples consisted of adjacent non-malignant omentum or peritoneum. All tumors were collected during primary cytoreductive surgery, prior to any chemotherapy treatment, and were stored as either fresh frozen (FF) or formalin-fixed paraffin-embedded (FFPE). These patients were separated into two groups, based on their overall survival. Patients who lived less than 3.5 years after their diagnosis were considered short-term (ST) survivors and patients who lived more than 5 years after diagnosis were considered long-term (LT) survivors (Table 1). Other clinical characteristics of patients are shown in Table 1. All patients received standard regimens of carboplatin and paclitaxel following cytoreductive surgery. More LT survivors received intraperitoneal (IP) chemotherapy than ST survivors (1 ST survivors, 5 LT survivors, p-value=0.042), Otherwise there were no differences in the use of bevacizumab or PARP inhibitor treatments between the two cohorts.
Recruitment	This was a retrospective study. All patients had FIGO stage II or higher High-grade serous ovarian cancer.
Ethics oversight	The ethics approval for this study was obtained through Washington University in St. Louis Institutional Review Board. Criteria for approval are met per 45 CFR 46.111 and/or 21 CFR 56.111 as applicable. All research conformed with the principles of the Declaration of Helsinki.

Note that full information on the approval of the study protocol must also be provided in the manuscript.

Field-specific reporting

Please select the one below that is the best fit for your research. If you are not sure, read the appropriate sections before making your selection.

☒ Life sciences ☐ Behavioural & social sciences ☐ Ecological, evolutionary & environmental sciences

Life sciences study design

All studies must disclose on these points even when the disclosure is negative.

Sample size	This was determined by feasibility and availability of specimens.
Data exclusions	We excluded 4 outlier samples for only our Differential expression RNA sequencing analyses. These 4 samples were removed from downstream analyses because of their distance from the other samples in the PCA after normalizing and batch correcting transcript counts.
Replication	Due to limited specimens, this study did not have additional specimens for reproducibility.
Randomization	N/A
Blinding	Blinding was not relevant to our study. The analysis was performed using an unsupervised approach.

Reporting for specific materials, systems and methods

We require information from authors about some types of materials, experimental systems and methods used in many studies. Here, indicate whether each material, system or method listed is relevant to your study. If you are not sure if a list item applies to your research, read the appropriate section before selecting a response.

Materials & experimental systems

n/a	Involved in the study	
☒	☐	Antibodies
☒	☐	Eukaryotic cell lines
☒	☐	Palaeontology and archaeology
☒	☐	Animals and other organisms
☐	☒	Clinical data
☒	☐	Dual use research of concern

Methods

n/a	Involved in the study	
☒	☐	ChIP-seq
☒	☐	Flow cytometry
☒	☐	MRI-based neuroimaging

Clinical data

Policy information about clinical studies

All manuscripts should comply with the ICMJE guidelines for publication of clinical research and a completed CONSORT checklist must be included with all submissions.

Clinical trial registration	N/A since this was a retrospective study
Study protocol	N/A
Data collection	The settings and locales of data collection is provided in the manuscript.
Outcomes	We pre-defined primary and secondary outcomes measures using clinically relevant endpoints. We assessed these measures using clinical criteria.
